# Pathological Anatomy

**Published:** 1861-01

**Authors:** John H. Packard

**Affiliations:** Philadelphia


### PATHOLOGICAL ANATOMY.

BY JOHN H. PACKARD, M.D., OF PHILADELPHIA.

I.—*Diseases of Brain.* By Dr. V. M. Beronius, of Fahlun, Sweden.

(1) *Abscess in the Right Cerebral Hemisphere.*—A. W., a merchant, aged forty-seven, had a profuse epistaxis in May, 1857, and from that time his health declined. He had various symptoms of cerebral disturbance, but nothing very marked until December twenty-third, when he was seized with vertigo while at the table. This left behind it headache, restlessness, insomnia, and hemiplegia of the left side, with a tendency to bedsores on the corresponding lower extremity; but these symptoms passed off in about five weeks. On March 5th, 1858, he had a severe epileptiform convulsion, chiefly affecting the left side, lasting five hours, and followed by coma; from the effects of this seizure he recovered in two weeks. After several such attacks, recurring about every fourth week, an unusually violent one, of a more apoplectic type, carried him off on the sixth of August.

The head was examined forty-eight hours after death. The membranes of the brain exhibited increased vascularity, but were completely free from exudation, and seemed somewhat œdematous. At the upper and back part of the right cerebral hemisphere, there was a slight depression in the cerebral substance, and the pia mater was thickened. At this spot, within two lines of the surface, was an abscess of the size of a small apple, containing a thick, greenish, pus-like fluid, with no particular smell. Two smaller cavities, of the size of walnuts, communicated with the first named by sinuous passages. Around the larger cavity there was a rather firm capsule, but the others were bounded by softened, reddish or yellowish brain-substance. In several of the blood-vessels of the brain atheromatous deposits were observed.

Dr. Beronius regards this case as one of capillary hemorrhage, or else of primary softening from embolia; the fatal result was in his opinion brought on either by congestion (?) or by cerebral paralysis.

(2) *Tumor of the Cerebellum.*—A woman, aged forty-five, healthy from childhood, began nine months before her admission into hospital to suffer from vertigo and singing in the ears, accompanied with progressive diminution of the power of vision, and uncertainty in walking. During her stay in the hospital these symptoms increased, and she became completely amaurotic in the right eye, and amblyopic in the left; her speech was faltering, she had difficulty in pronouncing words, and progressive inability to express herself. She never complained of any decided headache, but was extremely annoyed with buzzing in the right ear. Her gait became gradually more uncertain, and was attended with the peculiarity that at every step she lifted her feet very high, and felt as if the floor went up and down like waves; after walking a short time she began to totter, always turning round to the left, and falling down in that direction, if she was not supported. The power of walking visibly diminished, and complete paralysis would probably have ensued if erysipelas of the face had not supervened, of which, after three months' stay in the hospital, she died.

"On post-mortem examination a fibrous tumor of the size of a hen's egg was found beneath the tentorium, proceeding from the posterior surface of the petrous bone, and compressing the cerebellum" anteriorly on the right side.—(Translated from the *Hygiea*, January, 1860, for the *Dublin Medical Press*, October 10, 1860, by W. D. Moore, M.B., etc.)

II.—*Softening of the Medulla Spinalis, from the pressure of a Tumor.* By HR. MALMSTEN.

Charlotta Boll, aged forty-one, was admitted into the Seraphim Hospital, on the 23d of August, 1859. She had been healthy all her life, except occasional rheumatic pains, attacks of hysteria, and constipation. Two years previous to the above date, she received a blow on her breast, and has ever since been liable to pain and stitches in the right side. In September, 1858, she began to suffer from pains in the right shoulder and chest; and in the spring of 1859, a cough came on, with free expectoration. In May, she lost sensation in the right leg, and soon after in the left also; then both the lower limbs became powerless, with formication and occasional spasms. The anæsthesia extended to the abdomen, so that she voided her urine and fæces involuntarily, and even gave birth to a child almost without consciousness.

When admitted to the hospital, she was paraplegic, with tenderness over the ten superior dorsal spinous processes, as well as between the right scapula and the median line. Sensation and voluntary motion were lost, but reflex action could be produced, in the abdomen as well as in the lower limbs; she had a feeling of constriction around the trunk. Her skin was of an ash-gray color, and dry, but of normal temperature. Some bedsores had formed about the buttocks. Vomiting and diarrhœa supervened on the third of September, and carried her off in sixteen days.

On post-mortem examination, the chief lesion detected was as follows: "The dura mater and arachnoid of the medulla spinalis were healthy, but on opening the latter a tumor of nearly two inches in length was met with, of rather flattened form and loose consistence, which lay inclosed in a proper capsule, and extended from the second down to the fourth dorsal nerve; it had its seat on the right side of the spinal cord, and involved almost the entire of the right half of the latter; by this tumor the medulla spinalis was compressed and softened in this situation. The origins of the nerves were, particularly on the right side, compressed and partly atrophied. Both above and below the tumor the spinal cord appeared to be perfectly sound. The cerebro-spinal fluid was in rather more than normal quantity."

This tumor was lobulated, composed of fine connective tissue with numerous nuclei, the fibres being arranged in bundles overlaid "with small flat cells arranged like epithelium, almost all completely filled with large nuclei."

A curious lesion was observed in the *vermiform process* in this case; its point was strongly adherent to the parietal peritoneum, and the process here dilated to the size of a small plum, by purulent deposit. The tube was constricted above this enlargement, the mucous membrane lining which was ulcerated.—(Translated from the *Hygiea*, December, 1859, for the *Dublin Medical Press*, October 3, 1860, by W. D. MOORE, M.B., etc.)

### III.—*Epithelial Cancer of the Larynx.*

Primary cancer of the larynx is very rarely observed. In the present case, under the care of Dr. Alderson, in St. Mary's Hospital, London, the patient was a stout, plethoric man, aged forty-eight. He had complained of his throat for a year before his admission to the hospital, sometimes very seriously. A day or two before that time he had spit up some black matter, and subsequently some blood. He had much difficulty and stridor in respiration, and his breath was very fetid. Two days afterward, his dyspnœa became so alarming that tracheotomy was resorted to. Pneumonia set in after about a week, and carried him off in thirty-four days.

*Post-mortem examination.*—"On opening the pharynx from behind, a large ulceration was seen occupying the whole of the posterior wall of the larynx on its pharyngeal surface, and extending below and on the left side upon the mucous membrane of the membranous wall of the pharynx. Its surface was uneven and irregular, its color pale, and its margins well defined. At the parts where it had extended beyond the back of the pharynx, the ulceration was found to be raised slightly above the level of the healthy mucous membrane, and to involve the *mucous membrane* only. It could be seen from behind, too, that a tumor occupied the right side of the larynx; the thyro-aryteno-epiglottidean folds were much thickened by deposit, and ulcerated, (this not, however, reaching the epiglottis,) and the arytenoid muscle was completely destroyed, so that the interior of the larynx could be seen between the two disconnected arytenoid cartilages. On continuing the incision made at the operation, upward and downward, the interior of the larynx was found almost completely occupied by diseased structure, which seemed to proceed from the mucous membrane lining both alæ of the thyroid body; the surface presented was exceedingly irregular, uneven, and everywhere in a state of ulceration; the vocal cords were completely destroyed. A process of the disease had extended over the cricoid cartilage into the trachea posteriorly; another had pushed between the cricoid and thyroid cartilages on the left side, and formed a little tumor outside the larynx, about the size of half a hazel-nut, over which the fibres of the crico-thyroid muscle were stretched. Another tumor, rather larger, was found involving the thyroid body on the same side. *No* enlarged glands and *no* diseased bone were found. Matter taken from any part of the ulcerated surface showed, under the microscope, large epithelial scales of every variety of form, with large, and frequently double, nucleolated nuclei. Sections from different parts of the diseased mass gave these epithelial cells, with elastic tissue from the destroyed laryngeal structures. The trachea presented two or three slight ulcerations from the irritation of the canula; its entire mucous membrane was discolored—green, gray, etc.; the purulent matter in it and in the larynx was horribly fetid. The lungs were both in a state of gray hepatization, the right one most advanced; behind its lower lobe was an empyema, localized by adhesions; between this and one of the bronchial tubes which contained most pus, a tract of lung could be traced, yellower, softer, and more disorganized even than the rest, but no canal from one to the other by which matter had passed could be made out. The heart was healthy; kidneys rather large and fatty."—(*Lancet*, November 17, 1860.)

IV.—*Aneurism of Aorta behind the Aortic Valves.*—By DR. FINNELL.

A negro, aged twenty-five, who had seemed in perfect health previously, was found dead in his bed. At the autopsy there was found an aneurism of the aorta, as large as a walnut, just above the coronary arteries. The inner coat of the sac was ulcerated in many places, exposing the middle tunic. A very small orifice existed at one point, leading into the pericardium, which was very much distended with blood, partly clotted and partly fluid. About two inches above this aneurism was another, very much like it, but of smaller size. The aorta was abundantly atheromatous in the neighborhood.—(*Transactions of the New York Pathological Society*, in the *American Medical Times*, October 6, 1860.)

V.—*Aneurism of the Abdominal Aorta.* By DR. SANSOM.

A carpenter, aged thirty-eight, was admitted into King's College Hospital, May 18, 1860. He had been seized, three months previously, with a severe pain between the hypochondria; it came on suddenly as he was going to his work, and recurred at intervals. Some time afterward, he suffered from throbbing in the epigastrium, nausea, emaciation, and malaise; his urine was passed in small quantity, and with pain in the back.

On admission, the veins of the legs and of the surface of the abdomen were enlarged and tortuous. A distinctly pulsating tumor extended from the ensiform cartilage to near the umbilicus; another small movable swelling was felt in front of this one. In the tumor a thrill and a rasping bruit were perceived; there was an exaggerated curve in the lumbar region, and the ribs bulged very much. The urine was albuminous, with small waxy casts.

On the twenty-fourth he had an epileptic fit, and subsequently several very severe paroxysms of pain, so that chloroform and morphia had to be freely given. On the thirty-first, his face became pale, and his heart beat very feebly for about two hours, when he died.

At the autopsy, an aneurism as large as two fists, involving the aorta below the root of the superior mesenteric artery, was found to have burst into the cellular tissue between the layers of the meso-colon; the blood was traced down into the areolar tissue of the pelvis. The bodies of the last dorsal and first two lumbar vertebræ had been eroded. Over the surface of the aneurismal tumor ran the vena cava ascendens and the left renal vein, flattened out by stretching. The smaller swelling was found to be the pylorus.—(*Lancet*, Am. ed., September, 1860.)

VI.—*Empyema with Malignant Disease in the Mediastinum.* By DR. W. H. DICKINSON.

W. D., aged thirty-four, was admitted into St. George's Hospital on the 27th of June, 1860. He had been ailing about six months, with cough at first, to which were at length added dyspnœa, emaciation, and inability to lie on the left side. There was never any stitch or pain in the side. When admitted, his symptoms were those of chronic pleurisy and empyema on the right side; and paracentesis was advised, but not performed until the tenth day. Three days later, another small puncture was made, but he sank and died in about forty hours.

"*Autopsy*, thirty-five hours after death. Body somewhat emaciated; abdomen tympanitic. Thorax: There was a small opening far back, on the right side of the thorax, through which, with some trouble, a probe was passed into the pleural cavity; pus was exuding from this wound. The posterior mediastinum contained a large malignant tumor, of the hard, encephaloid variety; it filled all the space between the great vessels and the lung, and had attacked the latter. The whole tissue of the lung was perfectly disorganized, and presented a texture riddled with abscesses. This texture seemed to consist principally of the remains of bronchial tubes and vessels. There were no traces of malignant disease in it; it seemed perfectly useless as a breathing organ. The upper three-fourths of the right pleura seemed tolerably healthy in structure. It was distended by a very large quantity (more than a pint) of creamy pus. There were two openings into this cavity, toward its lower part: one, which seemed to have been made with the knife or trocar, was situated close to the upper border of the fifth rib; the other, larger and more irregular, was situated at the next lower intercostal space. They both communicated, though indirectly, with the external wound. The lower fourth of the pleura was adherent, and implicated, with the base of the lung and diaphragm, in a mass of malignant disease. The left pleura was healthy, with the exception of a few adhesions, as were the corresponding lung and the heart. In the arch of the aorta were a few loose glands infiltrated with malignant disease. The muscles around the wound were thickened, and two or three small abscesses existed there. Abdomen: A great part of the diaphragm was so infiltrated by the malignant disease that none of its natural tissue could be distinguished. The malignant mass reached down the spine, lying in front of the lumbar vertebræ, nearly as low as the sacro-vertebral angle. It was lobulated on its surface, and had much the appearance of a mass of diseased glands. The liver was large and soft; its anterior edge contained a single tubercle of malignant disease, about as large as a hazel-nut." The other organs, and the large vessels, were unaffected.—(*Lancet*, Am. ed., December, 1860.)

### VII.—*Contribution to the Pathological Anatomy of the Pancreas.* By DR. J. KLOB.

According to Dr. Klob, alterations of the pancreas, analogous to those seen in the *nutmeg* liver, may occur from the same causes, viz., from valvular disease of the heart, or from chronic pulmonary affections. They may also be induced by obstructions to the current in the vena cava or its ramifications within the liver.

At the outset, the pancreas is enlarged, gorged with liquids, and its sub-peritoneal areolar tissue is œdematous. On cutting into the gland, its lobules are found to be yellowish white, and the interstitial areolar tissue more or less reddened and infiltrated with serum; to this infiltration is due the enlargement of the organ. Microscopical examination detects in this interstitial texture numerous elements of new connective tissue. The acini and ducts exhibit no other alteration, except that there are fewer fatty granules in the acini than usual.

Somewhat later, the gland is seen to become smaller, while its consistence becomes considerably firmer. The yellowish-white or pale reddish-yellow color is more uniform; the new connective tissue, contracting as it organizes, increases the lobulation of the organ. Sometimes the compressed lobules are studded with molecular deposits of carbonate of lime; or we may observe fascicular crystals, probably of margaric acid, from the atrophied fatty tissue.

The whole organ may be involved in these changes, or they may be much more marked at certain points. Dr. Klob has seen them generally at the left extremity, more rarely in the body of the gland, and very rarely in the serous coat only. These partial degenerations are sometimes very marked, and then the affected part is replaced almost wholly by a fibrous mass, in which may be here and there detected the remains of the parenchyma, as well as cysts of variable size, formed probably from portions of parenchyma isolated by the contracting connective tissue.

Owing to these fibrous masses, the flow of bile through the common duct may be materially hindered, so as even to cause death.

Besides the lesions now described, interstitial hemorrhages may be induced by the same causes. Dr. Klob has seen them only in cases where the interference with the portal vein was very marked. Capillary apoplexies, of very small extent, would seem to be sometimes quite frequent in *cirrhosis* of the pancreas; in fact we may often observe pigmentary deposits or small bloody cysts in the contracted connective tissue.—(*Gazette Hebdomadaire*, October 12, 1860, from *Œsterreichische Zeitschrift für praktische Heilkunde*, 1860, No. 33.)

### VIII.—*On Movable Spleen.* By PROFESSOR ROKITANSKY.

Professor Rokitansky mentions three cases of this abnormity.

In the first, that of a young woman, aged twenty, who had undergone the Cæsarean section, the spleen was lying on the right ilium, its hilus upward; the organ was attached to a pedicle, consisting of the elongated and thinned pancreas and the splenic artery, the former being in three spiral coils around the latter; adhesions had formed between the spleen and the surrounding peritoneal surfaces.

In the second, that of a woman aged forty-six, the spleen was in the left iliac region, attached to a long pedicle and to the omentum.

In the third, that of an insane woman aged sixty-nine, the spleen was small, its capsule thickened and detached from its parenchyma; false membranes connected it with the sigmoid flexure and with the small intestines, as it lay at the left of the upper pelvic opening. It was attached to the gastro-splenic ligament, which was twisted upon itself, and split up into several strands; the splenic artery was elongated, contorted, and thin, at its division very narrow, and its branches obliterated and here and there ossified. The vein was altered in the same way. The pancreas was in every way normal.

Dislocation of the spleen generally occurs in females, as a consequence of enlargment of the organ, its ligaments being probably also relaxed. In descending, the spleen rotates, so as to twist its pedicle and narrow its vessels. This interfering with the supply of blood, may induce degeneration of the organ, or even the death of the patient.—(*Brit. and For. Med.-Chir. Review* for October, 1860, from *Zeitschrift der Gesellschaft der Aerzte*, 1860, No. 3.)

IX.—*Perforation of the Appendix Vermiformis by an Intestinal Concretion.* By A. M. VEDDER, M.D.

J. S., a boy aged eight years, had for several weeks complained occasionally of abdominal pain. September fifteenth, he had pain in the right side, extending high up; was chilly; and vomited. The next day his mother remarked the distention of his abdomen. The symptoms of peritonitis now developed themselves, with diarrhœa, and vomiting of greenish liquid; and in ten days the child died. Several days before death, fluctuation was observed in the right iliac region.

An autopsy showed patches of lymph over the intestines, and injection of the visceral peritoneum; adhesions existed, particularly near the right iliac region, where about a gill of pus was effused. On making pressure on the intestines, air bubbled up in this liquid. A hard, rough, acorn-like concretion, composed of phosphate and carbonate of lime and amorphous matter, with four small seeds as a nucleus, lay in this pus; it had evidently escaped through a ragged opening at the root of the vermiform appendix, around which, as well as within the appendix, the mucous membrane was thickened, softened, and highly injected.—(*American Medical Times*, November 3, 1860.)

X.—*Foreign Body in the Appendix Cœci.* By F. A. HOWE, M.D., of Newburyport.

A child, a little over four years of age, was observed by his friends, early in the past summer, to lose flesh and strength. In July, he had occasional attacks of nausea and vomiting; and on the twenty-seventh he swallowed a nickel cent. This was passed at stool the next day. Next morning he had fever, and peritonitis set in, to which he succumbed in sixty hours. He complained of no abdominal pain, but only of tenderness, throughout.

On post-mortem examination, there were evidences of acute peritonitis; in the pubic and left iliac regions the viscera were concealed by a thick layer of coagulated lymph, beneath which were several ounces of very fetid ichorous pus. "The appendix vermiformis was found much enlarged, and tightly twisted upon itself near its junction with the cœcum, while the other end extended quite to the left of the median line, and in this position it was bound by firm adhesions. Its appearance was very dark, nearly gangrenous. From an opening in its presenting surface, at least two lines in diameter, pus flowed freely, and seemed to have been conducted by the adhesions of the parts into the left iliac region." Within the appendix was found a hard concretion of the size of a large bean, formed concentrically about a small double oat. Its chemical composition is not stated. — (*Boston Medical and Surgical Journal*, October 18, 1860.)

XI.—*Invagination of five inches of the Ileum into the Colon.* By DR. E. S. THOMPSON.

A farmer's boy, aged fifteen, was admitted into King's College Hospital, London, for catarrh and slight rheumatic pains. A few hours afterward, he was seized with abdominal pain, vomiting, and purging of blood and mucus. A

tender and resistant mass, as large as a fist, was felt in the right iliac fossa. The symptoms became those of intussusception, with complete obstruction; on the seventh day collapse occurred, terminating in death on the tenth.

"*Post-mortem examination.*—The intestines were found to be loosely adherent to one another, and to the abdominal parietes, by recently effused lymph; the small intestines were congested and filled with fæcal matter; and the transverse colon was distended with flatus. On laying open the large intestine, a portion of the ileum, about five inches in length, was found invaginated within the cœcum and ascending colon, and tightly girt by the margins of the ileo-cœcal valve; the constricted part was highly congested, breaking down under the finger, and almost in a state of mortification."—(*Lancet*, Am. ed., October, 1860.)

XII.—*Obstruction of the Bowels from Adhesion to the Uterus.* By J. H. ALDRIDGE, M.D., of Southampton.

Mrs. S., aged fifty-seven, the mother of five children, had a severe attack of flooding in 1858. In February, 1859, symptoms of obstruction of the bowels came on, and became more and more aggravated until she died, on the tenth of April. Stercoraceous vomiting had existed for some weeks before the fatal issue, which seemed due to exhaustion.

*Autopsy, two days after death.*—The abdominal cavity alone was examined. The intestines were smeared with a yellowish, watery, offensive liquid, like that which had been vomited; but this may have escaped from the tube after death. The small intestines generally were greatly distended with gas; the ileum was highly inflamed throughout almost its entire length. Near its lower end were numerous dark-colored patches, of varying size, where the whole thickness of the wall was gangrenous. For several feet above the ileo-cœcal junction, the gut was so softened as to tear when one coil was moved upon another. Here the contents were tenacious and putty like, but on the upper portion the same watery matters were found as had been vomited during life. The colon was nearly empty.

It was therefore evident that the obstruction was low down in the ileum, and on removing the intestines with the pelvic viscera, it was found that the gut alluded to was firmly attached to the posterior surface of the uterus in such a manner as to form a right angle. The womb itself was in a state of cancroid degeneration, being converted into a mass of epithelial cancer.—(*Lancet*, Am. ed., December, 1860.)

XIII.—*On the Morbid Appearances in Death by Cold.* By FRANCIS OGSTON, M.D., of Aberdeen.

(1) A woman, forty-four years of age, a vagrant, was found dead by the roadside, on the morning of April 24th, 1860. She had been drinking whisky when last seen, the evening before; the night had been stormy, and there was snow on the neighboring high grounds at the time.

The body "was lying on its right side, the clothes wet, the arms by the sides, the knees approximated, the left knee fully, the right knee partly bent, the eyelids closed, the pupils natural, the mouth half open, the expression of the countenance placid, and the face, the outside of the left arm, and the fronts of both knees of a bright red hue, contrasting with the pallor of the rest of the surface, the back included, which everywhere showed well-marked cutis anserina.

"The appearances at the inspection which followed were as under: Scalp bloody; arachnoid membrane thickened and opaque; cerebral sinuses empty; blood in moderate quantity in the veins on the surface of the brain; cerebral mass paler, and containing less blood than usual; the cavities on each side of the heart, and the arteries and veins opening into them, distended to an unusual extent with blood, mostly in a fluid state, but mixed with a few red and colorless (fibrinous) clots. The blood in the heart and elsewhere nearly of the hue of arterial blood, and, unless when in bulk, after a few minutes' exposure to the air assuming wholly that appearance. Frothy mucus in the larynx and trachea; mucous coat of the trachea minutely injected; clusters of small petechiæ on the interior of the stomach, whose contents (milk curd) exhaled a spirituous odor; liver a good deal congested; the remaining viscera of the abdomen remarkably pale and bloodless; urinary bladder enormously distended."

(2) A man aged thirty, a street porter, traveling on foot on a very cold night, drank some whisky and ale at a tavern; he then got on a cart, to sleep while riding, and when the party stopped, having gone eight miles, he was found to be dead.

Dr. Ogston saw the body "in its original position on its back in the cart, with blood in moderate quantities in the nostrils, and on the face, hands, and clothes;" (he had been struck by one of his companions on the way;) "the joints rigid, the arms by the sides, and the knees half bent. Dusky-red patches were observed on the forehead, right side of the face, the chin, the outside of the right and inside of the left thighs, and on the insteps of both feet; with these exceptions, the front of the body, including the lips, markedly pale; dependent parts of the trunk and limbs livid.

"At the subsequent inspection, the following appearances presented themselves: Scalp pale; arachnoid membrane thickened and opaque; clear serum, rather abundantly under the arachnoid, in the cerebral ventricles, and at the base of the skull; excepting some fullness of the superior longitudinal sinus, the cerebral veins and sinuses contained but little blood; the mass of the encephalon unusually pale and bloodless; reddish frothy mucus in the trachea, and in considerable quantity in the bronchi; distinct œdema of the left lung; fluid blood in large quantities in the cavities on both sides of the heart, and in the arteries and veins connected with them; and the blood here as elsewhere, as in the previous case, presenting, except when in mass, a rich crimson arterial hue; the contents of the abdomen unusually pale and bloodless; a quantity of brownish fluid, smelling of beer, in the stomach; bladder enormously distended with clear urine."

Dr. Ogston remarks on the analogies observable between these cases and four others, published by him in the *British and Foreign Medico-Chirurgical Review* for October, 1855; but he very justly says that further observations are required to enable us to assume that like appearances are necessarily to be found in every case of death by cold, even in its least complicated form. The subject is not without interest in a pathological point of view, although it is chiefly important in connection with legal medicine.

